# Exploring the Effect of Thyroid Hormone on Serum Lipoprotein (a) Levels in Patients With Thyroid Hormone Dysfunction: A Systematic Review

**DOI:** 10.7759/cureus.66361

**Published:** 2024-08-07

**Authors:** Bo B Lwin, Apoorva Vashishta, Samreen Nishat, Isaac N Mueka, Maria U Hassan, Ravi K Pandey, Naiela E Almansouri

**Affiliations:** 1 Clinical Research, California Institute of Behavioral Neurosciences and Psychology (CIBNP), Fairfield, USA; 2 Clinical Research, NewYork-Presbyterian Queens, New York, USA; 3 Internal Medicine/Surgery, Richmond Gabriel University, Kingstown, VCT; 4 Internal Medicine, University of Tripoli, Tripoli, LBY

**Keywords:** sub-clinical hypothyroidism, lipoprotein(a), levo-thyroxine, risk factors cardiovascular diseases, overt hypothyroid, hyperthyroid, hypothyroid, thyroxine (t4), lp(a), thyroid hormones

## Abstract

Genetic variations among people mainly determine the blood levels of lipoprotein (a) (Lp(a)), and it is relatively stable throughout one’s lifetime. Nevertheless, there could still be other factors that control the Lp(a) level. Thyroid hormones are known to influence the serum lipid level by regulating the expression of key enzymes that are involved in lipid metabolism. Both hypo and hyperthyroidism are associated with changes in lipid levels. Even though thyroid hormone abnormalities have been shown to alter traditional lipid parameters like low-density lipoprotein (LDL-C), its influence on Lp(a) has not been established. This review aims to identify the relationship between Lp(a) and thyroid hormones by reviewing data from correlative studies and observing treatment-related Lp(a) level changes in thyroid disorders from interventional studies. We searched MEDLINE, Cochrane, and Google Scholar databases with predefined search criteria and search strategies for paper identification. Individual reviewers reviewed identified papers for selection. Finalized papers were reviewed for Lp(a) levels and their responses to treatment in patients with thyroid disorders to establish the relationship between Lp(a) and thyroid hormone. We concluded that the data were limited and sometimes contradicted one another to establish a clear relationship between Lp(a) and thyroid hormones. Even though correlative studies data showed strong indications that overt-hypothyroidism was associated with high Lp(a) levels, thyroid hormone replacement studies did not show any significant changes in Lp(a) levels compared to pre-treatment in patients with both overt-hypothyroidism and subclinical hypothyroidism. More clinical trials focusing on Lp(a) with longer periods of treatment and follow-up in thyroid patients are needed to establish the relationship between the two. The possibility of dose-related Lp(a) responses to thyroid hormone treatment should also be explored.

## Introduction and background

Lipoprotein (a) (Lp(a)) is a variant of low-density lipoprotein, a complex cholesterol complex with apolipoprotein (a) and apolipoprotein B-100. Lp(a) is mainly produced in the liver. Elevated blood Lp(a) levels are found to be associated with an increased risk of atherosclerotic cardiovascular disease (ASCVD) and stroke [1]. An estimated 20-25% of the world's population is believed to have elevated Lp(a) [2]. There has been recent interest in the risk of elevated Lp(a) levels since it is considered an independent and causal risk factor for ASCVD [3]. Lp(a) is pro-thrombogenic, pro-inflammatory, and pro-atherogenic. The thrombogenic potential of Lp(a) arises from the apolipoprotein (a) being similar to plasminogen. Apolipoprotein (a) competitively inhibits plasminogen conversion to plasmin and thus promotes thrombogenesis by interfering with the fibrinolysis of blood clots [4]. The atherogenic potential of Lp(a) comes from its ability to bind macrophages, increase foam cell formation, and increase cholesterol deposition in arterial walls to form atherosclerotic plaques. Therefore, an elevated level of serum Lp(a) is associated with atherosclerosis, increased occurrence of lipid plaques in the coronary arteries, and thrombosis in case of plaque rupture, which are all necessary ingredients to ASCVD events.

The blood levels of Lp(a) are determined mainly by genetics. The levels are not affected by diet, exercise, or lifestyle factors. Lp(a) levels are stable throughout life and adult levels are reached by the age of five years [5]. However, there could still be non-genetic factors that influence Lp(a) concentration. Thyroid hormone levels can be one of them. Thyroid hormone abnormalities - hypothyroidism (a low level of thyroid hormone in the body) and hyperthyroidism (an excessive thyroid hormone level in the body) - are well-known to be associated with dyslipidemia [6]. Thyroid hormones influence blood cholesterol levels by regulating key enzymes in lipid metabolism, cholesterol synthesis, and removal by the liver. The hormones increase the removal of cholesterol by increasing the expression of the low-density lipoprotein (LDL-C) receptors and decreasing the expression of proprotein convertase subtilisin/kexin type 9 (PCSK9) [7]. Hypothyroidism is one of the common causes of secondary hyperlipidemia. Even though the traditional cholesterol parameters like LDL-C and total cholesterol, increase in thyroid hormone deficiency, the Lp(a) level’s association with the hormone is not well established even though there are studies suggesting hypothyroidism to be associated with increased levels of Lp(a) or reduction of Lp(a) after treatment [8,9]. This systematic review was conducted to determine if there is an association between thyroid hormones and Lp(a) levels in patients with thyroid abnormalities.

## Review

The Preferred Reporting Items for Systematic Reviews and Meta-Analyses (PRISMA) 2020 guidelines were used to write this review [10].

Search and source strategy

PubMed, PubMed Central (PMC), Cochrane Library, and Google Scholar databases were searched for the relevant literature. Different search strategies with keywords - lipoprotein (a) and thyroid hormone - were used to identify papers in databases. In PubMed, a Boolean search using "AND" between the keywords was used. In addition, an advanced search and medical subject headings (MeSH) search were used to identify papers that might include synonyms related to the two keywords. No time limit was set in the PubMed search. All the papers identified through the three search strategies were included. A Boolean search strategy was also used in the Cochrane Library using "AND" between the keywords. The Harzing’s Publish or Perish application (Harzing, London, UK) was used to search for articles on Google Scholar. The search was limited to between 2014 and April 2024, with a maximum limit of 100 papers. The keyword search strategy was used in Google Scholar search with the search word: “lipoprotein (a) AND thyroid hormone.” The search criteria and results are shown in Table 1.

**Table 1:** Database search strategies and search result MeSH: medical subject headings

Database	Search method	Search range	Search criteria	Paper identified
Pubmed/Medline	Keyword search	1992 - April 2024	"Lipoprotein (a)" OR "Lp (a)" AND "thyroid hormone"	55
Pubmed	Advance search	1992 - April 2024	(((lipoprotein (a)) OR (Lp (a))) OR (lipoprotein (a)) AND (thyroid hormone)	110
Pubmed	MeSH search	1992 - April 2024	("Lipoprotein(a)"[Mesh] AND ("Lipoprotein(a)/blood"[Mesh] OR "Lipoprotein(a)/chemistry"[Mesh] OR "Lipoprotein(a)/metabolism"[Mesh] OR "Lipoprotein(a)/physiology"[Mesh])) AND ("Thyroid hormones/blood"[Mesh] OR "Thyroid hormones/chemistry"[Mesh] OR "Thyroid hormones/metabolism"[Mesh] OR "Thyroid hormones/physiology"[Mesh])	23
Google Scholar	Keyword search (Harzing's Publish or Perish - 100 maximum results)	2014 - 2024	Lipoprotein (a) AND thyroid hormone	100
Cochrane	Search manager	1995 - 2024	"Lipoprotein (a)" AND "thyroid hormone"	20
Total				308

Eligibility criteria

This review focused only on observational and clinical trial studies written and published in English. Included studies either assessed the Lp(a) level in patients with thyroid abnormalities (euthyroid (EU), overt-hypothyroid (OH), subclinical hypothyroid (SH) or hyperthyroid (HT)), or examined Lp(a) level changes before and after thyroid treatment. Only studies with human participants were included. However, congenital hypothyroid studies in newborns were excluded. Papers involving hormones other than thyroid and thyromimetics studies were excluded as well.

Studies Selection Process

The Endnote citation manager (Clarivate, London, UK) was utilized to combine and organize the identified papers transferred from different databases. Duplicate articles were removed using Endnote. Two reviewers screened the shortlisted articles by going through titles and abstracts independently and removed irrelevant papers for this review. The finalized papers were sought out for full-text paper retrieval. If full-text papers were not available, the authors were contacted to request them. Papers not received even after contacting the authors were removed from the study. After that, the full-text papers were reviewed for eligibility. Only eligible full-text articles that satisfied eligibility criteria were included in this review. In case of disagreements during the paper selection process, a third author got involved, and concerns were addressed through discussion and finalized via majority opinion.

Risk of Bias in Individual Studies

Appropriate quality appraisal tools for each study design were used for the risk of bias in finalized papers. The Newcastle-Ottawa Scale (NOS) quality assessment tool was applied for the quality assessment of cross-sectional observation studies [11]. A modified version of the Newcastle-Ottawa Scale (NOS) quality assessment was used for evaluating non-randomized pre- and post-treatment studies. The Cochrane Collaboration Risk of Bias Tool (CCRBT) was utilized in assessing the bias of randomized controlled studies [12]. Two reviewers assessed each paper independently. Any disagreements were resolved via discussion and mutual agreement or by consulting a third reviewer as a tie-breaker.

Only studies that had a low risk for bias with high-quality data were included in this study's analysis and interpretation. The set quality assessment score of 70% or higher was used in the NOS appraisal tool for paper acceptance. In addition, individual study variations in appraisal scores were also considered when finalizing our interpretations in this review.

Study Characteristics and Data Collection Process

The studies included in this review were cross-sectional studies that assessed the Lp(a) levels in patients with thyroid dysfunction and studies that observed the Lp(a) changes before and after treating thyroid abnormalities, for instance, Lp(a) changes in restoring euthyroid status in patients with hypo or subclinical hypothyroidism. Thyroid hormone normalization studies were included to determine the correlation between Lp(a) and thyroid hormones since the relationship between the two parameters was justifiable if correlative movements of the Lp(a) levels in response to thyroid hormone treatment were observed in the right direction.

Most of these studies in this paper assessed other lipid parameters besides Lp(a) lipid. However, only Lp(a) data were focused and extracted for this review. A pair of reviewers independently performed data abstraction for each assigned article, and collected data were checked and counter-checked for consistency and accuracy.

Outcome Measurements

In this review, the observed data for the outcome measurement included the correlation between Lp(a) levels and free thyroxine (FT4) levels, Lp(a) levels of thyroid patients compared to the control group, and the changes in Lp(a) levels before and after the restoration of euthyroid in patient groups. The correlative statistics data between Lp(a) and FT4 level were collected whenever available. If no correlative data were available in a paper, comparative data of FT4 level/thyroid functional status and Lp(a) between the patient and the control group or the serum Lp(a) changes pre- and post-treatment were observed. The relationship between Lp(a) and thyroid hormone levels was postulated if the serum Lp(a) levels were elevated in hypothyroid patients compared to control subjects or its levels were restored to a normal level after treatment or in subclinical or euthyroid patients whose FT4 levels should remain normal if the Lp(a) levels were observed to be similar to that of healthy control subjects. In the case of thyroid hormone replacement in subclinical hypothyroid patients, the Lp(a) levels should be slightly lowered or remain unchanged, depending on the extent of FT4 level changes following treatment. FT4 levels were the focus of this review since the FT4 level is a primarily measured parameter in thyroid function tests, and there is generally a good agreement between triiodothyronine (T3) and FT4 levels [13].

Results and Findings

**Figure 1:**
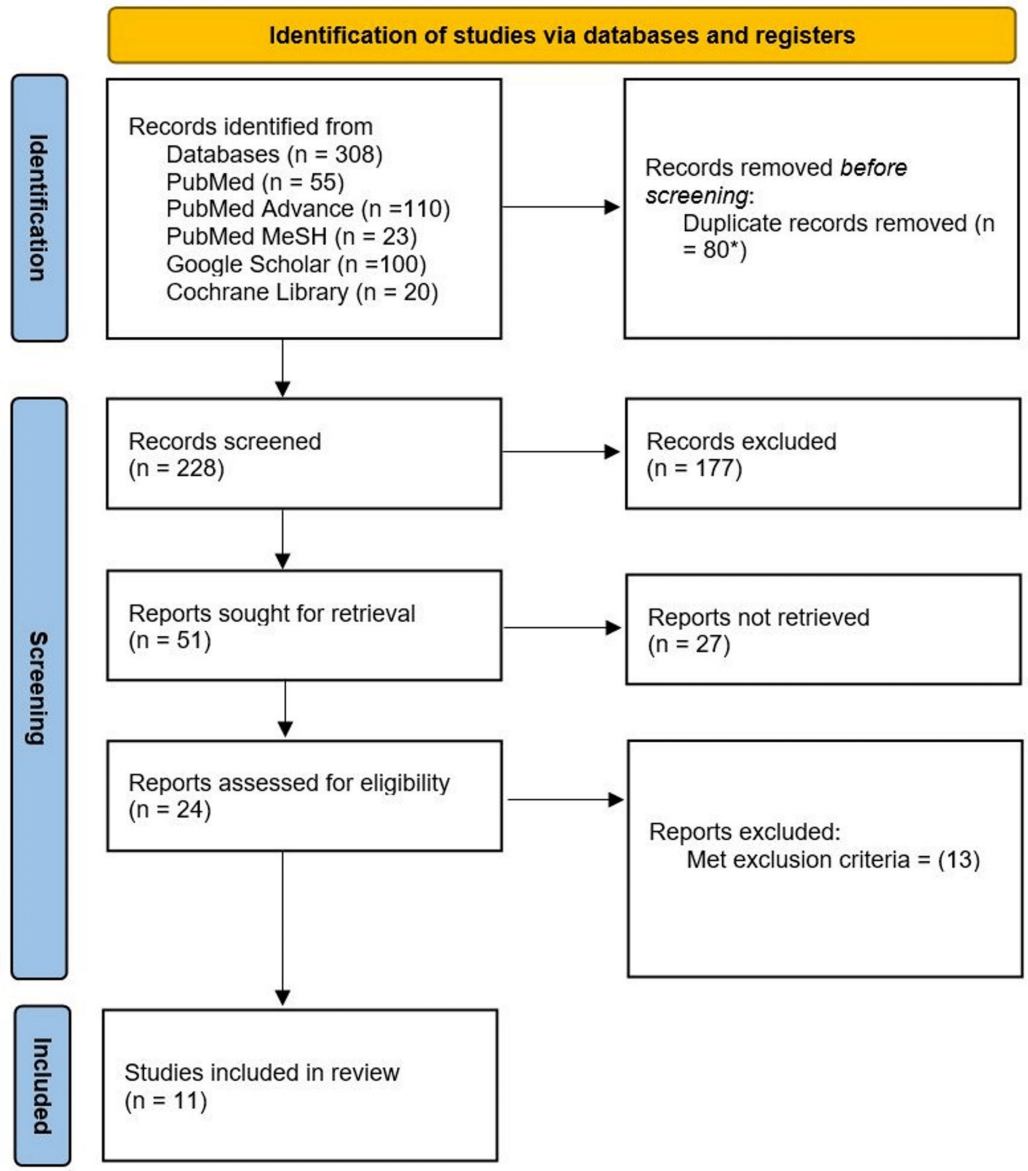
Preferred Reporting Items for Systematic Reviews and Meta-Analyses (PRISMA) flowchart *79 records were removed with EndNote citation manager automatically. One record was removed manually.

A total of 308 research papers were identified from the stated databases using different search strategies. Eighty out of 308 papers were duplicates. Those papers were removed using the Endnote citation manager automatic tool, but one was removed manually since the automatic tool could not identify the duplicate. After going through 228 remaining papers by titles and abstracts screening, 177 articles were removed since they were not related to this review or Lp(a) levels were not available or reported. Out of 51 remaining articles, 24 articles are available for full-text review. The remaining 27 articles were excluded since we could not retrieve them even after contacting and requesting from the authors. Thirteen articles were excluded since they did not satisfy our selection criteria. After the quality appraisal check, 11 articles were included in this systemic review. Figure 1 shows the PRISMA flow diagram [10].

Quality Assessment of Selected Studies

The quality assessments of the included studies were conducted using appropriate appraisal tools depending on the specific types of papers being reviewed.

Quality Assessment of Cross-Sectional Studies

The NOS quality assessment tool was utilized for evaluating cross-sectional studies. The tool allocates up to 3 points for selection, 1 point for comparability, and 3 points for outcome, with a maximum possible score of 7 points. Papers with a minimum of 5 out of 7 points (> 70%) were deemed acceptable. All three cross-sectional studies identified passed the quality assessment and were included in the review. Table 2 summarizes the results of the quality assessment for the cross-sectional studies.

**Table 2:** Quality assessment of cross-sectional observation studies The maximum possible score is 7 points. The passing score for the quality assessment is 5 points.

Study Paper	Selection			Comparability	Outcome			Result	
	Representativeness of the case	Selection of the control group	Sample size (over 50)	Comparability of case and control group (control for confounders)	Is the assessment of outcome appropriate?	Statistical test	Discussion of conclusion justified by result	Total score	Percentage
Bansal et al., 2016 [14]	1	1	1	1	1	1	1	7	100%
Yetkin et al., 2015 [15]	0	1	1	1	1	1	1	6	86%
Kaliaperumal et al., 2014 [16]	1	1	1	0	1	1	1	6	86%

Quality Assessment of Non-randomized Pre- and Post-thyroid Treatment Studies

The quality assessments of pre- and post-thyroid treatment studies were conducted using a modified version of NOS. This modified tool scores up to 4 points for selection, 1 point for comparability, and 4 points for outcome, with a maximum total of 9 points. Studies scoring at least 7 out of 9 points (> 78%) were included in this review. All six non-randomized studies met the quality assessment criteria and were therefore included. Table 3 summarizes the quality assessment results for the non-randomized pre- and post-treatment studies.

**Table 3:** Quality assessment of non-randomized pre- and post-thyroid treatment studies The maximum possible score is 9 points. The passing score for the quality assessment is 7 points.

Study Paper	Selection				Comparability	Outcome				Result	
	Representativeness of the cases	Sample size (over 50)	Ascertainment of exposure (thyroid hormone replacement or treatment)	Demonstration that outcome of interest was not present at the start of the study	Comparability of participants	Assessment of outcome	Was thyroid hormone treatment long or follow-up enough?	Discussion of conclusion justified by result	Statistical test	Total score	Percentage
Ito et al., 2013 [17]	1	0	1	1	1	1	0	1	1	7	78%
Adrees et al., 2009 [18]	1	1	1	1	1	1	1	1	1	9	100%
Ito et al., 2007 [19]	1	0	1	1	1	1	0	1	1	9	78%
Beyhan et al., 2006 [20]	1	1	1	1	1	1	1	1	1	7	100%
Yildirimkaya et al., 1996 [21]	1	0	1	1	1	1	0	1	1	7	78%
Engler et al., 1993 [22]	1	1	1	1	1	1	1	1	1	9	100%

Quality Assessment of Randomized Clinical Trials

CCRBT was utilized for the quality assessment of randomized controlled studies. Both studies identified were deemed to be at low risk of bias based on this assessment and were included in the review. Table 4 summarizes the quality assessment results for the randomized clinical trial studies. 

**Table 4:** Quality assessment of randomized clinical trials

Studies	Randomization process bias	Deviation from intended interventions bias	Missing outcome data bias	Measurement of the outcome bias	Selection of the reported result bias	Overall bias
Caraccio et al., 2002 [23]	Low	Low	Low	Low	Low	Low
Meier et al., 2001 [24]	Low	Low	Low	Low	Low	Low

Studies characteristics

Cross-Sectional Observation Studies

The results of the three cross-sectional studies demonstrated the correlation between Lp(a) and FT4 levels. The characteristics of these studies are summarized in Table 5.

**Table 5:** Characteristics of cross-sectional observation studies OH: overt-hypothyroid; SH: subclinical hypothyroid; EU: euthyroid; FT4: free thyroxine; Lp(a): lipoprotein (a)

No	Studies	Sample size				Lp(a) levels compared to the control group			Correlation between Lp(a) and FT4
		OH	SH	EU	Control	OH	SH	EU	
1	Bansal et al., 2016 [14]	130	0	0	130	Increase	NA	NA	Correlative
2	Yetkin et al., 2015 [15]	48	50	56	50	Increase	Similar	Similar	Correlative
3	Kaliaperumal et al., 2014 [16]	50	0	0	40	Increase	NA	NA	Correlative

Non-randomized Pre- and Post-thyroid Treatment Studies

Three out of six pre- and post-treatment studies demonstrated correlative changes in Lp(a) relative to FT4 levels, while the remaining three studies did not observe such results. The characteristics of those studies are summarized in Table 6.

**Table 6:** Characteristics of non-randomized pre- and post-thyroid treatment studies OH: overt-hypothyroid; SH: subclinical hypothyroid; FT4: free thyroxine; HT: hyperthyroid; Lp(a): lipoprotein (a); NA: not applicable; ND: not done (baseline comparison)

No	Studies	Sample size				Baseline Lp(a) level compared to control (if applicable)			Duration of thyroid hormone treatment	Lp(a) levels change after treatment			Changes in Lp(a) levels following treatment in relation to FT4 levels
		OH	SH	HT	Control	OH	SH	HT		OH	SH	HT	
1	Ito et al., 2013 [17]	18	18	0	0	NA	NA	NA	3 months	No change	No change	NA	Non-correlative
2	Adrees et al., 2009 [18]	0	56	0	56	NA	Increase	NA	18 months	NA	Decrease	NA	Correlative
3	Ito et al., 2007 [19]	13	26	0	0	NA	NA	NA	3 months	No change	No change	NA	Non-correlative
4	Beyhan et al., 2006 [20]	0	75	0	27	NA	ND	NA	4.2+/-1.01 months	NA	No change	NA	Non-correlative
5	Yildirimkaya et al., 1996 [21]	0	20	0	20	NA	Similar	NA	3 months	NA	Decrease	NA	Correlative
6	Engler et al., 1993 [22]	30	0	32	0	NA	NA	NA	2-11 months	Decrease	NA	Increase	Correlative

Randomized Pre- and Post-thyroid Treatment Studies

The findings from the two randomized pre- and post-treatment studies did not support a correlation between Lp(a) and thyroid hormone levels. The characteristics of these randomized clinical trials are summarized in Table 7.

**Table 7:** Characteristics of randomized clinical trials SH: subclinical hypothyroid; Lp(a): lipoprotein (a); FT4: free thyroxine

No	Studies	Sample size		SH group randomization		Treatment dosage and duration		Post-treatment Lp(a) level changes	Changes in Lp(a) levels following treatment in relation to FT4 levels
		SH	Control	Treatment	Placebo	Dosage	Duration		
1	Caraccio et al., 2002 [23]	49	33	24	25	67.5 ug/day	6-15 months	Not observed	Non-correlative
2	Meier et al., 2001 [24]	66	0	31	32	50-125 ug/day	48 weeks	Not observed	Non-correlative

Discussion 

There were 727 thyroid cases and 356 control subjects in the 11 studies we finalized for review.

Cross-Sectional Observation Studies

The three cross-sectional studies compared the Lp(a) levels in patients with thyroid diseases to healthy control patients.

Bansal et al. studied 130 overt-hypothyroid patients with 130 healthy aged and gender-matched individuals [14]. The study concluded that hypothyroid patients had a higher level of Lp(a) than control subjects. Their finding was statistically significant at p-value <0.001. They also concluded that Lp(a) and other non-conventional lipid parameters were more indicative of lipid status in hypothyroid patients than conventional parameters like total cholesterol (TC), triglyceride (TG), LDL-C, and high-density lipoprotein (HDL-C). The study found Lp(a) was positively correlated with thyroid-stimulating hormone (TSH) (R-value: 0.709, p-value: <0.001), but they did not report a correlation data between Lp(a) and FT4 levels.

Yetkin et al. studied Hashimoto patients with different thyroid functional statuses [15]. The study observed 48 overt-hypothyroid, 50 subclinical, and 56 euthyroid patients, all with positive Hashimoto thyroid antibodies. The study included 50 healthy subjects as a control group. Their study found that Lp(a) levels were elevated only in the overt-hypothyroid group, and the subclinical and euthyroid and control groups had similar Lp(a) levels compared to the control group. The study also found a negative correlation between Lp(a) and FT4 levels (R-value: -0.20, p-value: 0.003).

The study also conducted a sub-analysis on the status of Lp(a) excess in the participants, defined as an Lp(a) level over 30 mg/dl. The results showed that Lp(a) excesses were more common in the Hashimoto patient group (hypo, subclinical, and euthyroid patients) than in the control group. This finding suggests that autoimmunity may also influence Lp(a) metabolism and serum levels, even with normal TSH levels.

Kaliaperumal et al. observed 50 hypothyroid patients compared with 40 healthy subjects [16]. The study revealed a significant negative correlation between Lp(a) and FT4 (R-value: -0.303, p-value: 0.03). Their findings also implied that changes in thyroid hormone levels had a direct effect on serum Lp(a) levels.

The three cross-sectional observation studies collectively studied 334 thyroid patients compared to 220 healthy control subjects. Their findings supported that a low level of thyroid hormone in the body (overt-hypothyroidism) was associated with a high level of serum Lp(a). In the Yetkin et al. study, no Lp(a) level differences were found among subclinical, euthyroid, and control subjects [15]. This implied that Lp(a) levels depended on thyroid hormone levels but not on serum TSH levels (subclinical hypothyroid patients have elevated TSH levels). Based on these observational study data, the relationship between serum Lp(a) levels and thyroid hormone levels can be established.

*Non-randomized* *Pre- and Post-thyroid Treatment Studies*

This systemic study included six pre-post studies that observed Lp(a) level changes in patients with thyroid abnormalities after treatment. 

Ito et al. 2013 studied 18 overt-hypothyroid and 18 subclinical hypothyroid patients [17]. The study found no significant changes in Lp(a) levels in both overt-hypothyroid and subclinical hypothyroid patients after three months of thyroid hormone therapy. Even though FT4 levels went up significantly, no Lp(a) level changes were observed. The study did not include a control group; therefore, there were no baseline Lp(a) level comparisons between the disease and control groups.

Adrees et al. observed the effect of levothyroxine replacement on lipid levels in 56 subclinical hypothyroidisms with 56 control individuals [18]. The duration of replacement was 18 months. The study data revealed an increase in Lp(a) level in patients with subclinical hypothyroidism compared to the control group and its normalization after 18 months of levothyroxine treatment. The reviewers noticed that the FT4 level in the subclinical hypothyroid group was significantly lower than the control group even though they were within the normal range, which could have explained the high Lp(a) level in the subclinical group. Normalizing Lp(a) levels after 18 months of levothyroxine treatment favors the idea that thyroid hormones negatively correlate with serum Lp(a) Levels. 

Ito et al. 2007 observed thyroid hormone replacement in 13 overt-hypothyroid and 26 subclinical hypothyroid patients [19]. The study did not observe a change in Lp(a) levels with hormone replacement in either of the two groups. The duration of the thyroid hormone treatment in this study was three months and no baseline Lp(a) comparison between the two groups was included in the study data.

Beyhan et al. included 75 subclinical hypothyroid cases and 27 control subjects in their study [20]. The duration of thyroid replacement was 18.2+/-4.4 weeks. The study found no significant Lp(a) level changes after the treatment. Therefore, the authors concluded that thyroid hormone did not affect Lp(a) levels. The study provided no comparative Lp(a) data of the patient group with the control group at baseline or post-treatment.

Yildirimkaya et al. studied 20 subclinical hypothyroid and 20 control subjects [21]. The study found a slightly higher Lp(a) level in the subclinical hypothyroid group compared to the control group. They also found a 25% decrease in Lp(a) level after three months of treatment with low-dose levothyroxine. The authors concluded that there might be a dose-related Lp(a) response to thyroid replacement and that low-dose (25ug/day) thyroid hormone replacement could lower Lp(a) levels but higher doses might have the opposite effect. 

Engler et al. was the only study that included analysis of Lp(a) relationship with both hypothyroid and hyperthyroid patients before and after treatment [22]. Sixty patients participated in the study, with 30 hypothyroid cases and 32 hyperthyroid cases (two cases with opposite changes). The study found a significant improvement in Lp(a) levels after thyroid hormone treatment in hypothyroid patients. The reversal of Lp(a) levels was observed during the treatment of hyperthyroidism. The study also observed that the effects of thyroid hormone on Lp(a) were more pronounced during hyperthyroidism treatment than in hypothyroidism treatment. This study also examined the levels of T3. The post-treatment variations in T3 levels exhibited a strong correlation with FT4 levels in both hypothyroid and hyperthyroid groups. Additionally, a reciprocal relationship between T3 levels and Lp(a) levels was observed. The study suggested that thyroid hormone exerts a direct suppressive effect on apolipoprotein (a) synthesis. Since the Lp(a) level depends on the amount of apolipoprotein (a) availability, thyroid hormone could affect the Lp(a) level by modulating the biosynthesis of apolipoprotein (a) at the transcriptional or translational level.

While the correlative cross-sectional studies provided compelling evidence of the relationship between Lp(a) and thyroid hormone, the interventional pre- and post-treatment studies did not yield strong enough evidence to establish a clear relationship. Three out of the six studies observed overt-hypothyroid patients [17,19,22], and only one of them, Engler et al., observed a decrease in Lp(a) levels with thyroid hormone treatment, potentially indicating a relationship between the two [22]. The other two studies did not find a similar relationship. It's worth noting that the treatment duration and follow-up of those two studies were relatively short (three months), and it's possible that there was not enough time for a significant hormonal effect on serum Lp(a). Five studies included subclinical hypothyroid patient groups. Two out of five studies observed a reduction in Lp(a) with thyroid hormone replacement [18,21], whilst the other three studies did not achieve a similar outcome [17,19,20]. Of the two studies that observed the changes, one study followed patients for a longer duration of treatment (18 months), while the other study highlighted the use of low-dose treatment for their positive findings. Again, the other three studies that failed to observe Lp(a) changes implemented shorter duration and higher doses of thyroid hormone treatment.

Based on the data from the pre- and post-treatment studies, the reviewers were unable to establish a clear relationship between thyroid hormone and Lp(a). There were conflicting findings in Lp(a) changes after hormone replacement therapy in hypothyroid patients. An increase in Lp(a) levels after treatment of hyperthyroidism was observed in one study, but no other studies were available to confirm the finding. However, the reviewers believed that the variation in the treatment duration, follow-up periods, and treatment dosage across the studies might have obscured the true relationship between Lp(a) and thyroid hormone.

Randomized Clinical Trials

Caraccio et al. was a randomized placebo-controlled clinical trial [23]. The study included 49 subclinical hypothyroid patients and 33 control subjects. The patients were randomized between levothyroxine and placebo and were reassessed for their lipid profile six months after stable euthyroidism was achieved. The study did not find Lp(a) changes before and after treatment in both placebo and hormone groups. The treatment group's average levothyroxine dose to gain euthyroidism was 67.5 ug daily. The baseline lipoprotein profile of subclinical hypothyroid patients compared to the control group showed elevated Lp(a) levels, but it was not statistically significant. The study found that patients with increased Lp(a) levels were frequently associated with a history of type 2 diabetes mellitus and/or coronary heart disease. Therefore, the study authors concluded that serum Lp(a) levels reflect a genetic variation rather than a reduced thyroid hormone action.

Meier et al. was a double-blinded placebo-controlled trial in which 63 subclinical hypothyroid patients were assessed for changes in lipid profile in response to hormone replacement for 48 weeks [24]. The study did not find a significant Lp(a) lowering after 48 weeks of treatment after bringing participants to a euthyroid state. The mean dose of levothyroxine was 85.5+/-4.3 ug/day in this study.

Based on the two clinical trials, it was concluded that thyroid hormone treatment in subclinical hypothyroid patients did not reduce Lp(a) levels. However, none of those studies include overt-hypothyroid patients. Since the goal of treatment in subclinical hypothyroid patients is to normalize TSH, the changes in thyroid hormone levels in these patients may be minimal. As a result, any corresponding changes in Lp(a) levels might be too small to detect. The dose-dependent treatment effect on Lp(a) levels suggested by Yildirimkaya et al. 1996 study should also be explored more in future studies.

Limitations of the study

This study has a few limitations. Only two out of 11 studies in this review were randomized clinical trials. Most studies focused on lipid profiles as a whole rather than specifically on Lp(a) levels. Consequently, the potential genetic variations of Lp(a) levels were not controlled in the majority of the studies despite the Lp(a) data being observed and analyzed. Additionally, the review did not include information on race and ethnicity, which could be significant factors influencing variations in Lp(a) levels. The disparities in treatment duration and follow-up periods across the reviewed articles may also have a confounding effect on the interpretation and conclusion of this review.

## Conclusions

Based on this review, the authors found insufficient data to establish the relationship between Lp(a) and thyroid hormone. While cross-sectional studies observed the relationship between serum Lp(a) and thyroid hormone levels, the findings from the pre- and post-thyroid treatment studies were in discord to reach the same conclusion. More Lp(a) specific studies properly controlled for genetic variations with extended follow-up periods are needed to detect the relationship between the two.
